# Impact of the COVID-19 pandemic on mortality and loss to follow-up among patients with dementia receiving anti-dementia medications

**DOI:** 10.1038/s41598-024-58316-z

**Published:** 2024-04-05

**Authors:** Hyuk Sung Kwon, Wonjae Sung, Keun U. Park, Seung Hyun Kim, Seong-Ho Koh, Jae-Sung Lim, Hojin Choi

**Affiliations:** 1 Department of Neurology, Hanyang University Guri Hospital, Hanyang University College of Medicine, Guri, Republic of Korea; 2 Department of Neurology, Hanyang University College of Medicine, Seoul, Republic of Korea; 3 Claim Data Analyst, Seoul, Republic of Korea; 4 Department of Neurology, Asan Medical Center, 88 Olympic-ro 43-gil Songpa-gu, Seoul, 05505 Republic of Korea

**Keywords:** Dementia, COVID-19, National Health Insurance Service, Alzheimer’s disease, Health care, Neurology, Risk factors

## Abstract

The coronavirus disease 2019 (COVID-19) pandemic has profoundly impacted vulnerable groups, such as patients with dementia. We examined changes in mortality and loss to follow-up in patients with dementia using data from the Korean National Health Insurance Service research database. Patients with dementia who visited a medical institution with a recorded dementia-related diagnostic code, including Alzheimer’s disease, and who received anti-dementia medication between February 2018 and January 2020 were included in this study. We divided patients with dementia receiving anti-dementia medications into two cohorts: those newly diagnosed with dementia between February 2018 and January 2019 (n = 62,631) and those diagnosed between February 2019 and January 2020 (n = 54,494). Then, we conducted a one-year follow-up of their records, tracking the cohort diagnosed between February 2018 and January 2019 from February 2019 to January 2020, as well as the cohort diagnosed between February 2019 and January 2020 from February 2020 to January 2021. There was a significant increase in follow-up loss among patients newly diagnosed with dementia during the COVID-19 outbreak, from 42.04% in 2019 to 45.89% in 2020. Female sex, younger age, fewer comorbidities, diagnosis of dementia at the Department of Neurology or Psychiatry, and higher income were associated with decreased follow-up loss and mortality. This study highlights the importance of paying extra attention to patients with dementia receiving anti-dementia medications, particularly during pandemics, given their increased risk of loss to follow-up.

## Introduction

The first cases of coronavirus disease (COVID-19) were reported in Wuhan, China in December 2019^1^. It entered Korea in January 2020 and spread rapidly in February 2020 after a woman with a fever, later confirmed as having COVID-19 infection, participated in multiple events of the Shincheonji Church in the city of Daegu^2,3^. With the declaration of a pandemic by the World Health Organization in March 2020, the Korean government put into effect a quarantine policy, which included social distancing^2,4^. It caused neuropsychological and social problems that reduced physical activity, nutritional intake, and economic activities among older adults^5^.

Global mortality rates have increased since the spread of COVID-19. Older adults with pre-existing health conditions such as cardiovascular disease accounted for the majority of these deaths^6^. As patients with dementia are more vulnerable to worsening neuropsychiatric symptoms and behavioral changes after social isolation^7^, the impact of the COVID-19 pandemic has raised concerns among individuals living with dementia. More specifically, individuals with dementia may encounter challenges in remembering and comprehending the safeguarding procedures or public health guidance provided to them and have difficulty living alone without help or accessing reliable information about COVID-19^8^. In addition, restricting outdoor activities may lead to disconnection from society.

However, little research has been conducted on how the COVID-19 pandemic has affected the mortality or management of individuals with dementia and which patient subgroups are more vulnerable to its effects. Therefore, identifying vulnerable subgroups for mortality or loss of management is a crucial issue when developing strategies for managing pandemics, especially with the increasing demand for critical care and limited healthcare resources.

In the current study, patients diagnosed with dementia receiving anti-dementia treatment as of February 2020 and monitored before and after treatment were investigated to identify differences in mortality and loss to follow-up. We also aimed to investigate whether differences in the follow-up loss rates occurred across various patient characteristics using the National Health Insurance (NHIS) database in South Korea.

## Results

**Figure 1:**
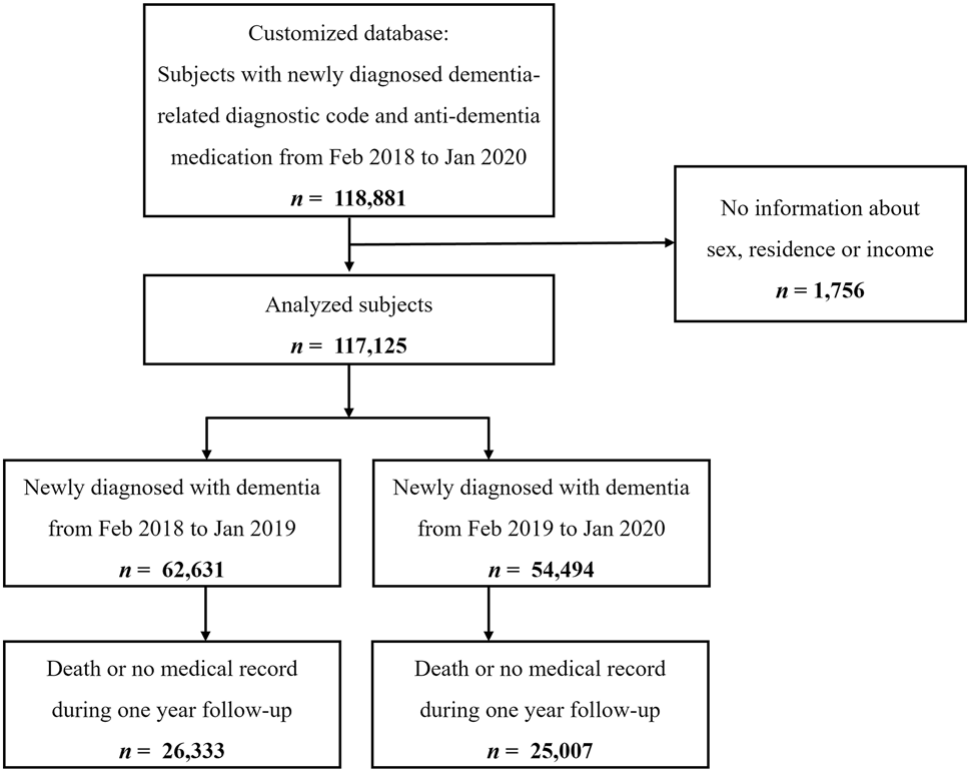
Identification of participants from the National Health Insurance Database included in the analysis.

Between February 2018 and January 2020, 117,125 patients with a dementia-related diagnostic code who received anti-dementia medication had at least one healthcare visit. Among them, 62,631 were diagnosed in 2018 (from February 2018 to January 2019) and 54,494 were diagnosed in 2019 (from February 2019 to January 2020). During the one-year follow-up, 18,607 (15.89%) patients died, and 51,340 (43.83%) did not visit medical institutions with dementia-related diagnostic codes (Fig. 1 and Table 1). Follow-up loss increased from 42.04% among patients diagnosed with dementia receiving anti-dementia medications in 2018 to 45.89% in 2019.

**Table 1:** Characteristics of patients with newly diagnosed dementia in 2018 and 2019.

	Total	Year of diagnosis		*p-*value^b^
		2018^a^	2019^a^	
Total number	117,125 (100.0)	62,631 (100.0)	54,494 (100.0)	
Age				< 0.001
< 50	704 (0.6)	424 (0.7)	280 (0.5)	
50–59	3369 (2.9)	1983 (3.2)	1386 (2.5)	
60–69	11,870 (10.1)	6626 (10.6)	5244 (9.6)	
70–79	38,964 (33.3)	21,492 (34.3)	17,472 (32.1)	
≥ 80	62,218 (53.1)	32,106 (51.3)	30,112 (55.3)	
Sex, female	74,870 (63.9)	39,823 (63.6)	35,047 (64.3)	0.009
CCI score				< 0.001
0	2,178 (1.9)	1,036 (1.7)	1,142 (2.1)	
1–2	34,663 (29.6)	18,202 (29.1)	16,461 (30.2)	
≥ 3	80,284 (68.6)	43,393 (69.3)	36,891 (67.7)	
Comorbidities in CCI				
Chronic kidney disease	6299 (5.38)	3235 (5.17)	3064 (5.62)	< 0.001
COPD	8172 (6.98)	4401 (7.03)	3771 (6.92)	0.481
Dyslipidemia	81,957 (69.97)	43,765 (69.88)	38,192 (70.08)	0.444
Diabetes mellitus	58,574 (50.01)	31,135 (49.71)	27,439 (50.35)	0.029
Hypertension	85,617 (73.10)	45,902 (73.29)	39,715 (72.88)	0.116
Cerebrovascular disease	37,446 (31.97)	21,328 (34.05)	16,118 (29.58)	< 0.001
Department				< 0.001
Neurology or Psychiatry	81,625 (69.69)	43,109 (68.83)	38,516 (70.68)	
Other	35,500 (30.31)	19,522 (31.17)	15,978 (29.32)	
Residence				< 0.001
Metropolitan	48,097 (41.06)	26,081 (41.64)	22,016 (40.40)	
Non-metropolitan	69,028 (58.94)	36,550 (58.36)	32,478 (59.60)	
Income, quintile				0.4121
< 40%	42,845 (36.58)	22,805 (36.41)	20,040 (36.77)	
40–60%	13,972 (11.93)	7,473 (11.93)	6,499 (11.93)	
≥ 80%	60,308 (51.49)	32,353 (51.66)	27,955 (51.30)	
Death during one-year follow-up^c^	18,607 (15.89)	9,851 (15.73)	8,756 (16.07)	0.115
Follow-up loss during 1 year^d^	51,340 (43.83)	26,333 (42.04)	25,007 (45.89)	< 0.001

Data are presented as number (%), unless otherwise indicated.

*CCI* Charlson–Romano comorbidity index, *COPD* chronic obstructive pulmonary disease.

^a^2018: 2018-02-01–2019-01-31; 2019: 2019-02-01–2020-01-31.

^b^Pearson’s chi-square was used.

^c^Death within the next year.

^d^Death or no medical records in the following year.

The demographics, comorbidities, diagnosis department, and residence of the incident cases diagnosed in 2018 and 2019 are shown in Table 1. The total number of patients with dementia receiving anti-dementia medications decreased from 62,631 in 2018 to 54,494 in 2019, increased with age, and was higher among female patients. Among patients with dementia receiving anti-dementia medications, 73.1% had hypertension, 50.0% had diabetes mellitus, and 70.0% had dyslipidemia-related diagnostic codes. A total of 69.7% of patients were diagnosed with dementia at the Department of Neurology or Psychiatry. Finally, more than half of the patients belonged to the highest income group (80%).

**Figure 2:**
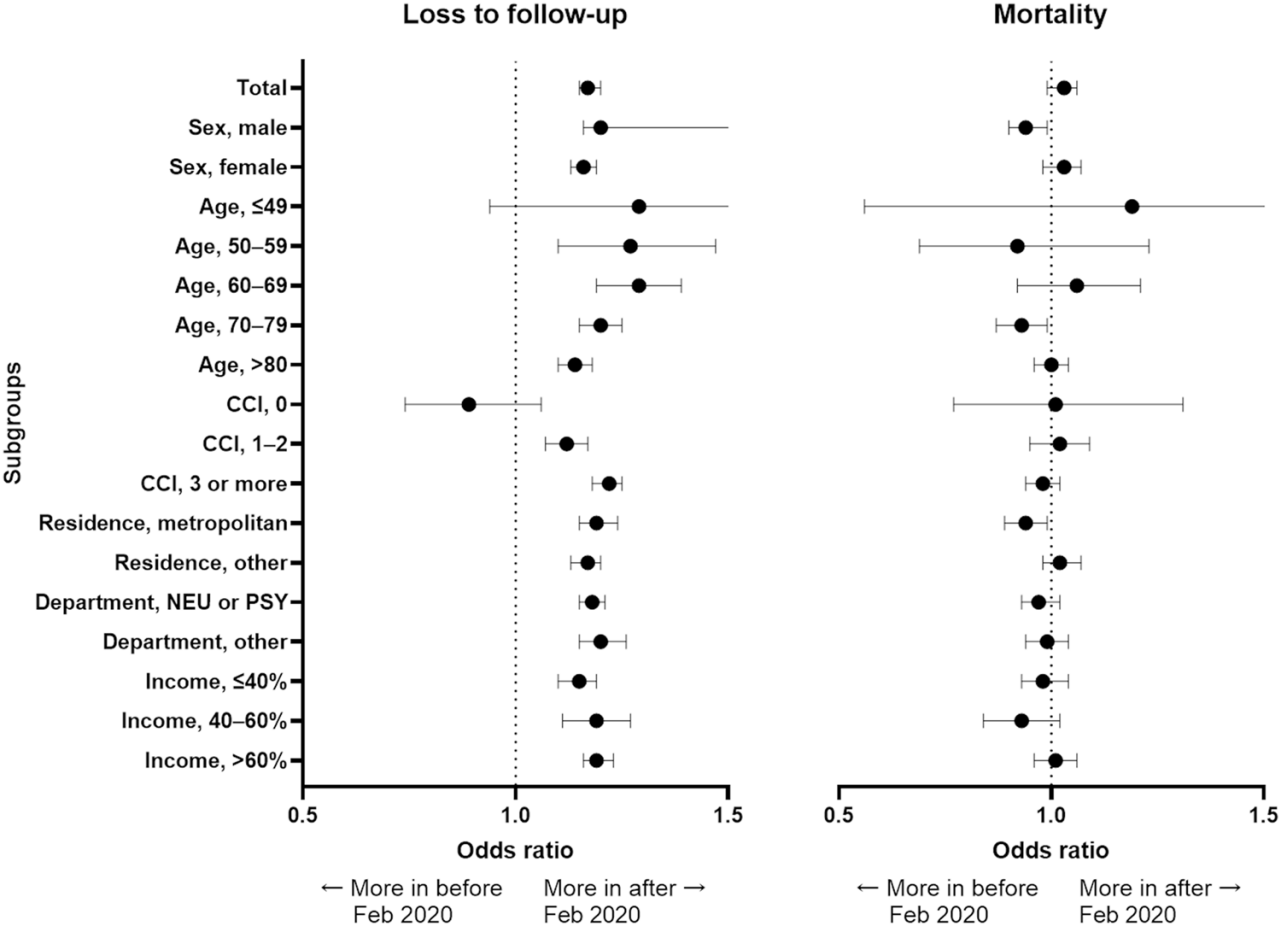
Comparing follow-up loss and mortality before and after February 2020 according to subgroups. The error bar indicates a 95% confidence interval. *CGI* Charlson-Romano comorbidity index, *NEU* Neurology *PSY* Psychiatry.

Logistic regression analysis was performed to identify potential risk factors for loss to follow-up (Table 2) and mortality (Table 3) over one year. Patients who were diagnosed with dementia while receiving anti-dementia medications in 2019 and followed during the COVID-19 pandemic showed higher follow-up loss (OR 1.17, 95% CI 1.15–1.20) than those diagnosed in 2018. However, mortality was not significantly different between patients diagnosed with dementia receiving anti-dementia medications in 2018 and in 2019 (Fig. 2).

**Table 2:** Multivariable logistic regression analysis of potential risk factors for follow-up loss in patients with dementia.

	Unadjusted OR	Adjusted OR	*p-*value
Diagnosed year, 2019 compared to 2018	1.17 (1.14–1.20)	1.17 (1.15–1.20)	< 0.001
Sex, male	1.07 (1.04–1.09)	1.09 (1.06–1.12)	< 0.001
Age, group			
< 50	1.09 (0.94–1.26)	0.91 (0.76–1.06)	0.2443
50–59	0.91 (0.85–0.98)	0.86 (0.80–0.92)	< 0.001
60–69	0.89 (0.86–0.93)	0.89 (0.85–0.92)	< 0.001
70–79	0.85 (0.83–0.87)	0.88 (0.86–0.90)	< 0.001
≥ 80	1 [Ref]	1 [Ref]	
CCI			
0	0.74 (0.68–0.81)	0.73 (0.67–0.80)	< 0.001
1–2	0.87 (0.84–0.89)	0.90 (0.87–0.93)	< 0.001
≥ 3	1 [Ref]	1 [Ref]	
Residence, metropolitan	0.85 (0.83–0.87)	0.90 (0.88–0.92)	< 0.001
Medical department, Neurology, or Psychiatry	0.58 (0.57–0.60)	0.59 (0.58–0.61)	< 0.001
Income level			
< 40%	1.11 (1.08–1.14)	1.07 (1.04–1.10)	< 0.001
40–60%	1.07 (1.04–1.12)	1.07 (1.03–1.11)	0.0004
≥ 60%	1 [Ref]	1 [Ref]	

Data are presented as odds ratios (95% confidence interval). Logistic regression analysis was used, *p* for multivariate models. Adjusted for age group, sex, CCI score, residence, comorbidity, classification of medical institutions, medical department, and income level. *CCI* Charlson–Romano comorbidity index.

**Table 3:** Multivariable logistic regression analysis of potential risk factors for mortality in patients with dementia.

	Unadjusted OR	Adjusted OR	*p-*value
Diagnosed year, 2019 compared to 2018	1.03 (0.99–1.06)	0.99 (0.96–1.02)	0.5190
Sex, male	1.75 (1.70–1.81)	2.10 (2.03–2.17)	< 0.001
Age, group			
< 50	0.17 (0.12–0.25)	0.09 (0.06–0.13)	< 0.001
50–59	0.27 (0.24–0.31)	0.18 (0.16–0.21)	< 0.001
60–69	0.35 (0.33–0.37)	0.27 (0.26–0.29)	< 0.001
70–79	0.48 (0.46–0.49)	0.45 (0.43–0.46)	< 0.001
≥ 80	1 [Ref]	1 [Ref]	
CCI			
0	1.07 (1.03–1.11)	0.89 (0.78–1.01)	0.0711
1–2	0.99 (0.94–1.04)	0.83 (0.80–0.87)	< 0.001
≥ 3	1 [Ref]	1 [Ref]	
Residence, metropolitan	0.95 (0.92–0.98)	1.04 (1.00–1.07)	0.0283
Medical department, Neurology, or Psychiatry	0.47 (0.46–0.49)	0.47 (0.46–0.49)	< 0.001
Income level			
< 40%	1.07 (1.03–1.11)	1.12 (1.08–1.16)	< 0.001
40–60%	0.99 (0.94–1.04)	1.05 (0.99–1.10)	0.0927
≥ 60%	1 [Ref]	1 [Ref]	

Data are presented as odds ratios (95% confidence interval). Logistic regression analysis was used, *p* for multivariate models. Adjusted for age group, sex, CCI score, residence, comorbidity, classification of medical institutions, medical department, and income level. *CCI* Charlson-Romano comorbidity index.

## Discussion

In this study, we observed a significant increase in patient loss to follow-up among newly diagnosed patients with dementia receiving anti-dementia medications following the aggressive spread of COVID-19. However, there was no significant difference in the mortality rate between the periods before and after February 2020. Female sex, younger age, fewer comorbidities, diagnosis of dementia at the Department of Neurology or Psychiatry, and high income were associated with decreased follow-up loss and mortality. Moreover, living in metropolitan areas was associated with a reduced loss to follow-up.

The observed increase in follow-up loss among patients with dementia is consistent with findings of previous studies that reported a decrease in outpatient visits or hospital admissions following the COVID-19 pandemic^9,10^. The COVID-19 pandemic has significantly impacted follow-up loss in patients with dementia, with essential factors including concerns about the risk of COVID-19 infection, restriction of outdoor activities, exacerbated cognitive decline due to prolonged isolation, impaired communication with non-cohabitating caregivers, reduced accessibility to non-COVID-19 medical services at public healthcare facilities, and challenges in hospital visits stemming from stringent COVID-19 prevention measures. The stringent healthcare policies implemented by South Korea in response to the early surge in COVID-19 cases have led to restrictions on outdoor activities among the South Korean population. Continuous isolation is linked to accelerated cognitive deterioration, hindering patients’ ability to keep hospital appointments and to access necessary care^11^. The pandemic has also strained relationships between patients and caregivers, leading to missed appointments and a lack of healthcare management. Moreover, the prioritization of COVID-19 treatments in public healthcare facilities has delayed care for patients with dementia, and strict infection prevention protocols have made it difficult for these patients to receive in-person care. These factors collectively underscore the complex impact of the pandemic on healthcare access and continuity of care for patients with dementia.

However, it was unexpected that there were no significant differences in mortality rates before and after February 2020, considering that individuals with dementia are known to have a higher risk for the acquisition and mortality from COVID-19 infection^12^. It is possible that the lack of difference in mortality rate was due to the short duration of the observation period. However, diverse policies for dementia, including the nationwide expansion of infrastructure such as dementia support centers since 2017, socioeconomic support for patients with dementia and caregivers, and the establishment of national dementia helplines, may have contributed to these results^13–15^. The overwhelming effect of South Korea's healthcare system on the surge in COVID-19 cases may be another factor^16^.

Higher mortality was observed in patients with dementia receiving anti-dementia treatment in those with older age, male sex, and higher CCI scores, which was similar to that in the general population with COVID-19 infection^4^. We have previously reported that the community management rate may decrease in patients with dementia if they are older, have a higher comorbidity burden, live in non-metropolitan areas, or have a low income^13^. In the current study, we observed higher rates of both follow-up loss and mortality among patients with the following characteristics: male sex, older age, higher CCI, and low income. Therefore, it is imperative to focus on these patient groups during the pandemic.

In this study, patients diagnosed with dementia in the Department of Neurology or Psychiatry had better follow-up and mortality outcomes. Approximately 70% of the patients were diagnosed with dementia in the Department of Neurology or Psychiatry. If dementia is diagnosed in other departments, it is possible that cognitive decline has already progressed significantly or that there are other underlying diseases. However, this difference remained significant even after adjusting for age and comorbidities. Specialized training on dementia, appropriate education for patients and caregivers, and easier access to resources such as dementia support centers may have influenced these results.

This study had several limitations. First, it was impossible to confirm the prevalence of COVID-19 in patients with dementia. Second, it was not feasible to confirm dementia diagnoses due to the study's reliance on national health claims data, which lack detailed clinical histories and exam results. Patients often visit multiple healthcare providers, resulting in a variety of dementia-related diagnostic codes. This diversity, coupled with unclear dementia subtypes, precluded the disclosure of subtype-specific characteristics. Furthermore, relying solely on NHIS diagnostic codes may lead to potential overestimation. Consequently, our study focused on patients with dementia who were prescribed anti-dementia medications, aligning with findings from previous research^14,17,18^. Approximately 75% of individuals diagnosed with dementia were prescribed anti-dementia medications, representing a majority of the total diagnosed population^14,19^. Furthermore, a recent study reported that among patients diagnosed with dementia and receiving anti-dementia medications, a significant proportion was diagnosed with Alzheimer’s disease (66.5%), and there was a high female ratio (69.8%) within the cohort^18^. This approach may have unintentionally excluded some patients with dementia, particularly those with vascular dementia or frontotemporal dementia, who did not receive anti-dementia medications. Third, regression analyses were conducted by merging the two cohorts due to constraints in the available data. Consequently, we could not determine the changes in risk factors before and after the pandemic. If the proportions of subgroups in the two cohorts were analyzed and the differences were compared, it would provide a better understanding of the impact of the COVID-19 pandemic on each subgroup. Fourth, the short follow-up period and the lack of a pre-pandemic parallel cohort may not comprehensively account for the natural progression of the conditions under study or pre-existing trends in follow-up loss or mortality.

In conclusion, the mortality rate of patients with newly diagnosed dementia receiving anti-dementia treatment in South Korea did not change before and after February 2020. The loss to follow-up significantly increased after February 2020. Nevertheless, male patients who were older, had a higher number of comorbidities, diagnosed with dementia in departments other than neurology or psychiatry, and had a low income may have required more attention to maintain follow-up.

## Methods

### Data source

This study was conducted using anonymous customized research data extracted from the NHIS Database between February 1, 2017, and January 31, 2021. This database is primarily based on the Korean NHIS, a single government insurer that covers approximately 97% of the Korean population and supports Korean hospitals and nursing facilities. The customized database is representative of the transmission data provided by anonymous health insurance and long-term care insurance data^20^. The database provides healthcare utilization information for both in- and outpatients, including patient demographics, diagnoses, comorbidities, and prescribed medications. The Korean Classification of Disease (KCD), 5th–7th editions, and a modification of the International Classification of Disease and Related Health Problems, 10th revision, were used to code the diagnoses. The NHIS coding system was used to collect demographic data (including age, sex, and income) and accompanying diagnostic codes, including diabetes (E10–14), chronic obstructive pulmonary disease (J44), chronic kidney disease (N18), dyslipidemia (E78), stroke (I60–64), hypertension (I10–15), and depression (F32, F33, and F34.1). The type of anti-dementia drug (donepezil, galantamine, rivastigmine, or memantine) was also extracted, and the pharmaceutical prescription codes for the anti-dementia drugs are described in Supplementary Table 1.

### Ethical approval

This study was approved by the Institutional Review Board of Hanyang University Guri Hospital (2022-04-040) and registered with the Clinical Research Information Service (CRIS) under registration number KCT0008217. All personal information in the NHIS database was de-identified, and the requirement for informed consent was waived by the ethics committee of Hanyang University Guri Hospital. Furthermore, all methods were performed in accordance with the relevant guideline (STROBE checklist) and regulations.

### Study population

All individuals in the customized research database visited a medical institution with a recorded dementia-related diagnostic code and anti-dementia medication from February 2018 to January 2020 (Fig. 1). Dementia was identified in the claims data based on *KCD-5, 6,* or* 7* codes. Patients with dementia were defined as those with a history of outpatient visits or admissions based on dementia-related diagnostic codes and anti-dementia drug use. Dementia-related diagnostic codes were F00 (Dementia in Alzheimer’s disease), F01 (Vascular dementia), F02 (Dementia in other diseases classified elsewhere), F03 (Unspecified dementia), G30 (Alzheimer’s disease), G31.00 (Behavioral variant frontotemporal dementia), G31.01 (Semantic variant primary progressive aphasia), G31.02 (Nonfluent primary progressive aphasia), G31.03 (Logopenic primary progressive aphasia), G31.04 (Primary progressive aphasia), and G31.82 (Dementia with Lewy bodies). We established a washout period commencing in 2002. We excluded all patients who had documented visits to healthcare facilities with a dementia-related diagnostic code before February 2018.

Patients with dementia receiving anti-dementia medications were divided into two groups: (1) newly diagnosed with dementia between February 2018 and January 2019, and (2) newly diagnosed with dementia between February 2019 and January 2020 (Fig. 1). The records were followed for one year until January 2020 to January 2021. No medical records with dementia-related diagnostic codes during this period were considered as “follow-up loss,” while death was considered as “mortality.” Mortality data were obtained from the death dates recorded within the NHIS database.

### Charlson Comorbidity Index (CCI)

We used the ICD-10 version of the CCI, which includes 17 diagnostic categories of acute myocardial infarction, congestive heart failure, peripheral vascular disease, cerebral vascular accident, dementia, pulmonary disease, connective tissue disorder, peptic ulcer, liver disease, diabetes mellitus, diabetes mellitus with complications, paraplegia, renal disease, cancer, metastatic cancer, severe liver disease, and HIV^21^. As all participants had dementia, the other 16 diagnostic categories were weighted and calculated as the CCI. The weighted values and corresponding ICD-10 codes are listed in Supplementary Table 2.

### Statistical analysis

The annual numbers of patients with dementia in 2018 (from February 2018 to January 2019) and 2019 (from February 2019 to January 2020) were identified and followed for one year to determine mortality and follow-up loss (Fig. 1). Descriptive analyses were conducted to verify the demographic features of the two cohorts. All patients with dementia receiving anti-dementia medications were divided according to age (5 groups: < 50, 50–59, 60–69, 70–79, and ≥ 80), sex, and income (quintiles) and compared. Categorical variables are expressed as percentages or frequencies. Logistic regression analyses were performed to assess the relationships between various parameters (including year of diagnosis, age, sex, CCI score, residence, classification of the medical department, and income level) and follow-up loss or mortality. Age, sex, CCI score, residence, comorbidities, classification of medical institutions, classification of diagnosed medical departments, and income level were adjusted for as potential confounders. All statistical analyses were performed using SAS system version 9.4 (SAS Institute Inc., Cary, NC, USA) and Python 3.9.11 with the SciPy library, and *p* < 0.05 was considered statistically significant.

### Supplementary Information

Supplementary Tables.

## Data Availability

The datasets generated or analyzed during the current study are available from the National Health Insurance Sharing Service (NHIS) at https://nhiss.nhis.or.kr. Upon an individual re-searcher’s dataset request, NHIS provides customized data to the researcher.
