# Supplementary material for: Impact of the COVID-19 pandemic on mortality and loss to follow-up among patients with dementia receiving anti-dementia medications
Source: Sci Rep. 2024 Apr 5;14:7986. doi: 10.1038/s41598-024-58316-z (PMC10994909; doi:10.1038/s41598-024-58316-z)
Supplement: Supplementary file 1 — Supplementary Tables. [file 41598_2024_58316_MOESM1_ESM.pdf]

Table S1. Pharmaceutical prescription codes for antidementia drugs.

Antidementia drug	Drug classification code	Pharmaceutical prescription codes
Donepezil	119	148601ATB, 148601ATD, 148602ATB, 148602ATD, 148603ATB, 643401ATD, 643402ATD
Rivastigmine	119	224501ACH, 224503ACH, 224504ACH, 224505ACH, 224506CPC, 224507CPC, 224508CPC
Galantamine	119	385203ACR, 385203ATR, 385204ACR, 385204ATR, 385205ACR, 385205ATR
Memantine	119	190001ALQ, 190001ATB, 190002ASY, 190030ASY, 190031ALQ, 190003ATD, 190004ATD, 190004ATB

Table S2. Diagnostic categories in Charlson comorbidity index, weights, and corresponding ICD-10 codes

Condition	Weights	ICD-10 codes
Acute myocardial infarction	1	I21, I22, I252
Congestive heart failure	1	I50
Peripheral vascular disease	1	I71, I790, I739, R02, Z958, Z959
Cerebral vascular accident	1	I60, I61, I62, I63, I65, I66,G450, G451, G452, G458, G459, G46, I64, G454, I670, I671, I672, I674, I675, I676, I677 I678, I679, I681, I682, I688, I69
Pulmonary disease	1	J40, J41, J42, J44, J43, J45, J46, J47, J67, J44, J60, J61, J62, J63, J66, J64, J65
Connective tissue disorder	1	M32, M34, M332, M053, M058, M059, M060, M063, M069, M050, M052, M051, M353
Peptic ulcer	1	K25, K26, K27, K28
Mild liver disease	1	K702, K703, K73, K717, K740, K742, K746, K743, K744, K745
Diabetes mellitus	1	E109, E119, E139, E149, E101, E111, E131, E141, E105, E115, E135, E145
Diabetes mellitus with complications	2	E102, E112, E132, E142 E103, E113, E133, E143 E104, E114, E134, E144
Paraplegia	2	G81 G041, G820, G821, G822
Renal disease	2	N03, N052, N053, N054, N055, N056, N072, N073, N074, N01, N18, N19, N25
Cancer	2	C0, C1, C2, C3, C40, C41, C43, C45, C46, C47, C48, C49, C5, C6, C70, C71, C72, C73, C74, C75, C76, C80, C81, C82, C83, C84, C85, C883, C887, C889, C900, C901, C91, C92, C93, C940, C941, C942, C943, C9451, C947, C95, C96
Metastatic cancer	3	C77, C78, C79, C80
Severe liver disease	3	K729, K766, K767, K721
HIV	6	B20, B21, B22, B23, B24
